# Dexmedetomidine sedation reduces atrial fibrillation after cardiac surgery compared to propofol: a randomized controlled trial

**DOI:** 10.1186/s13054-016-1480-5

**Published:** 2016-09-21

**Authors:** Xu Liu, Kai Zhang, Wei Wang, Guohao Xie, Xiangming Fang

**Affiliations:** Department of Anesthesiology and Intensive Care Medicine, the First Affiliated Hospital, School of Medicine, Zhejiang University, 79 Qingchun Road, 310003 Hangzhou, China

**Keywords:** Atrial fibrillation, Cardiac surgery, Dexmedetomidine, Propofol, Sedation

## Abstract

**Background:**

Atrial fibrillation occurs frequently in patients following cardiac surgery and can be a cause of increased morbidity and mortality. The use of dexmedetomidine to prevent atrial fibrillation is unclear. The present study was designed to evaluate the effect of dexmedetomidine sedation on the incidence of atrial fibrillation after cardiac surgery.

**Methods:**

Upon arrival to the intensive care unit (ICU), cardiac surgery patients without prior atrial fibrillation or flutter were randomized to receive either dexmedetomidine (0.2–1.5 μg/kg/h) or propofol (0.3–3 mg/kg/h) open-label titrated to a target Richmond agitation-sedation scale of 0 to -3. Our primary endpoint was the incidence of postoperative atrial fibrillation, and the secondary end points were the length of ICU stay, length of hospital stay, and hospital costs.

**Results:**

Atrial fibrillation occurred in 6 of 44 patients (13.6 %) in the dexmedetomidine group compared to 16 of 44 patients (36.4 %) in the propofol group (odds ratio = 0.28; 95 % confidence interval, 0.10, 0.80; *P* = 0.025). The median (interquartile range) length of ICU stay in the dexmedetomidine group was significantly lower than in the propofol group (2.9 (2.4–3.5) vs 3.5 (2.7–4.5 days, *P* = 0.008), with a trend toward a decrease in median hospital costs (86,367 vs 77,874 Chinese yuan; *P* = 0.068). The incidence of hypotension was higher in the dexmedetomidine group than in the propofol group (25/44 (56.8 %) vs 13/44 (29.5 %); *P* = 0.017).

**Conclusions:**

Dexmedetomidine sedation reduced the incidence of new-onset postoperative atrial fibrillation and shortened the length of ICU stay in patients after cardiac surgery compared to propofol sedation. Dexmedetomidine treatment was associated with more episodes of hypotension.

**Trial registration:**

chictr.org.cn: ChiCTR-IPR-16008231, retrospectively registered: April 6, 2016.

This trial was not prospectively registered due to a lack of importance applied to trial registration.

**Electronic supplementary material:**

The online version of this article (doi:10.1186/s13054-016-1480-5) contains supplementary material, which is available to authorized users.

## Background

Atrial fibrillation (AF) is the most common complication and the most common arrhythmia detected following cardiac surgery [1–4]. The incidence of AF in patients after cardiac surgery has been reported to range between 15 and 50 % [5–7], with patients undergoing valve surgery being at the greatest risk [8]. In addition, AF most commonly occurs within 4 days postoperatively [5, 8]. Postoperative AF is associated with a prolonged hospital stay, higher costs, and increased morbidity and mortality [4–8].

Although a number of prophylactic strategies for the prevention of postoperative AF have been proposed, they are not routinely implemented in many clinical centers [9, 10], including ours. The major reasons for non-use include a lack of convincing evidence, potential risks associated with current drug therapies, and complexity of some prevention regimens [11, 12]. Sedatives and analgesics are routinely administered to most patients undergoing cardiac surgery to reduce anxiety and pain [13, 14]. Dexmedetomidine has been used as a safe and efficacious sedative agent in patients undergoing cardiac surgery [15, 16], without pro-tachyarrhythmic or negative inotropic effects [17]. Moreover, dexmedetomidine is associated with decreased incidence of postoperative supraventricular and ventricular tachyarrhythmia and has been found to terminate supraventricular tachycardia in pediatric patients [18–20]. Recently, two retrospective studies also showed that dexmedetomidine sedation might decrease atrial arrhythmia in adult patients after cardiovascular surgery [21, 22]. It appears that dexmedetomidine sedation would be a good choice for the prevention of postoperative AF. However, previous studies have been subject to selection bias and many confounders due to the observational design. Furthermore, the aforementioned studies did not directly compare dexmedetomidine with propofol, which is used in current standard practice for sedation after cardiac surgery.

We designed and implemented a prospective randomized controlled trial (RCT) to determine whether dexmedetomidine reduces the incidence of postoperative AF while effectively sedating mechanically ventilated patients after cardiac surgery, when compared to propofol sedation.

## Methods

### Patient population

This prospective randomized controlled clinical trial was approved by the Ethics Committee of the First Affiliated Hospital of Zhejiang University and registered at chictr.org.cn (ChiCTR-IPR-16008231). This study was conducted in the First Affiliated Hospital of Zhejiang University between January 2015 and December 2015. Written informed consent was obtained from the patient or next of kin before enrollment. The inclusion criteria were as follows: age ≥18 years, elective cardiac surgery with cardiopulmonary bypass (CPB), admitted to intensive care unit (ICU) while intubated and ventilated, and lack of prior AF or flutter before receiving sedation in the ICU. Patients were excluded when they had at least one of the following characteristics at arrival in the ICU: heart rate <50 beats per minute, atrioventricular conduction block of grade II or III (unless a pacemaker had been installed), mean arterial pressure (MAP) <55 mmHg (despite appropriate intravenous volume replacement and vasopressor treatment), acute severe neurological disorder, propofol or dexmedetomidine allergy or other contraindications. In addition, patients who had received two or more sedatives within 24 h postoperatively were also excluded.

### Randomization, masking and intervention

Patients received standard anesthesia including induction with midazolam and sufentanil and paralysis with cisatracurium and maintenance with sevoflurane, propofol and sufentanil. During CPB, the administration of sevoflurane was stopped. The bypass circuit was primed with 1000 mL of lactated Ringer solution, 100 mL of 5 % sodium bicarbonate and 200 mL of 20 % mannitol. Prior to aortic cannulation, heparin was adjusted to maintain an activated clotting time longer than 450 seconds. The pump flow rates ranged from 1.8 to 2.4 L/min/m^2^. The core temperature was controlled at 32 to 34 °C using a heat exchanger in the bypass circuit. After surgery, the patients were transferred to the ICU. Upon arrival at the ICU, the patient’s blood pressure, electrocardiograms and oxygen saturation were monitored. The patients were randomized in a 1:1 ratio to receive sedation with either propofol (control) or dexmedetomidine according to the random number table.

Dexmedetomidine or propofol was continuously infused without a loading dose. Sedation levels were evaluated with the Richmond agitation-sedation scale (RASS), which ranges from −5 (unarousable) to 4 (combative). An assessment of RASS was performed every 2 h or more often if required (e.g., if the patient’s condition changed). The intravenous infusion speed of dexmedetomidine (≤1.5 μg/kg/h) or propofol (≤3 mg/kg/h) was adjusted to maintain RASS values between 0 and -3 [23, 24]. When hypotension (MAP <65 mmHg) and/or bradycardia (heart rate <60 beats/minute) lasted longer than 5 minutes, the infusion of the sedative was stopped until the patient exhibited blood pressure and heart rate within an acceptable range. The infusion of the sedative was stopped before extubation at the discretion of the attending physicians.

Sufentanil was continuously infused (usually at a rate of 0.06 μg/kg/h) for intravenous analgesia and discontinued until a total dose of 200 μg had been administered. Pain was assessed using the visual analog scale (VAS) (range 0 (no pain) to 10 (maximal pain)). If the VAS score was >3 [25], additional analgesia (morphine or tramadol) was provided.

### Outcome measures

The primary outcome was the incidence of AF within 96 h of surgery. Postoperative AF was defined as no consistent P waves before each QRS complex and an irregular ventricular rate [3]. Each patient underwent continuous electrocardiographic monitoring for at least 96 h after surgery. The investigators recorded the time of AF onset when they detected AF on the electrocardiogram displayed on the bedside monitor, and a twelve-lead electrocardiographic recording was performed to confirm the rhythm if necessary. AF episodes lasting longer than 5 minutes were recorded [3].

Adverse events recorded during the ICU stay included: bradycardia (heart rate <60 beats/minute for >5 minutes [26]), hypotension (MAP <65 mmHg for >3 minutes), and postoperative nausea/vomiting and delirium (identified with a confusion assessment method for the ICU). Acute kidney injury was diagnosed according to Acute Kidney Injury Network (AKIN) criteria: an absolute increase in serum creatinine ≥26.4 μmol/L, percentage increase in serum creatinine ≥50 % (1.5-fold from baseline), or urine output <0.5 ml/kg/h for >6 h within 48 h after surgery [27]. The intubation time (time from ICU admission to the time of extubation), length of ICU stay, length of (postoperative) hospital stay and hospital fees were also recorded.

### Statistical analysis

Sample size determination was based on an expected 35 % occurrence of postoperative AF in the propofol group and an expected 10 % occurrence of AF in the dexmedetomidine group [11, 22, 28]. At a significance level of 0.05 with a power of 0.80, the resulting sample size was 40 patients in each group. Anticipating a dropout rate of approximately 10 %, a sample size of 45 patients in each group was considered to be appropriate. Data were described using descriptive statistics and presented as the median (interquartile range) unless indicated otherwise. All continuous variables were analyzed using the Mann-Whitney *U* test due to the relatively small numbers. Categorical variables were compared using the Fisher exact test. Kaplan-Meier curves were generated for postoperative AF. SPSS (SPSS 16.0 for Windows; SPSS, Chicago, IL, USA) and GraphPad Prism 5.04 (GraphPad Software, La Jolla, CA, USA) were used for statistical analyses. A *P* value <0.05 (two-sided) was regarded as statistically significant.

## Results

**Fig. 1:**
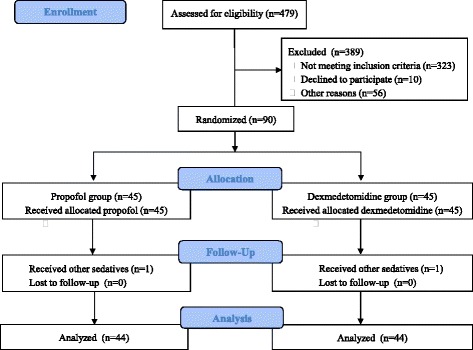
Consort flow diagram of the study participants

A total of 479 patients were assessed for eligibility after cardiac surgery with CPB (Fig. 1). After 389 patients were excluded, 90 patients were randomly assigned to two groups: 45 patients received propofol and 45 patients received dexmedetomidine. One patient in each group was also infused with the other sedative within 24 h after surgery. Therefore, 44 patients in each group were included in the analysis. There were no differences between the groups in baseline demographic and surgical characteristics (Table 1).

**Table 1:** Baseline demographic and surgical characteristics of the study population

Characteristics	Propofol (n = 44)	Dexmedetomidine (*n* = 44)	*P* value
Age, years	56.5 (49.3–62.0)	53.0 (46.0–63.0)	0.314
Female gender	30 (68.2)	23 (52.3)	0.191
Body mass index, kg/m^2^	21.8 (20.0–25.1)	22.4 (20.8–24.6)	0.607
Hypertension	13 (29.5)	13 (29.5)	>0.999
Diabetes	5 (11.4)	6 (13.6)	>0.999
Smoking history	10 (22.7)	18 (40.9)	0.108
Chronic obstructive pulmonary disease	2 (4.5)	4 (9.1)	0.676
Left ventricular ejection fraction, %	65.0 (57.0–71.0)	65.0 (56.8–71.0)	0.975
New York Heart Association class			0.561
I	2 (4.5)	2 (4.5)	
II	28 (63.6)	29 (65.9)	
III	12 (27.3)	13 (29.5)	
IV	2 (4.5)	0 (0)	
Preoperative use of β-blockers	5 (11.4)	1 (2.3)	0.202
Preoperative use of digoxin	20 (45.5)	20 (45.5)	>0.999
Type of operation			0.616
Mitral valve surgery	8 (18.2)	11 (25.0)	
Aortic valve surgery	15 (34.1)	15 (34.1)	
Mitral + aortic valve surgery	13 (29.5)	8 (18.2)	
CABG+ valve surgery	1 (2.3)	3 (6.8)	
Other cardiac surgery	7 (15.9)	7 (15.9)	
Cardiopulmonary bypass time, minutes	68.8 (53.8–93.6)	73.5 (55.7–85.1)	0.635
Cross-clamp time, minutes	47.3 (36.6–67.1)	50.7 (34.2–62.4)	0.892
Defibrillation after reperfusion	14 (31.8)	18 (40.9)	0.507
Temporary pacemaker insertion^a^	7 (15.9)	6 (13.6)	>0.999

Data are presented as the median (interquartile range) or number (%). ^a^Ventricular pacing. *CABG* coronary artery bypass graft

**Fig. 2:**
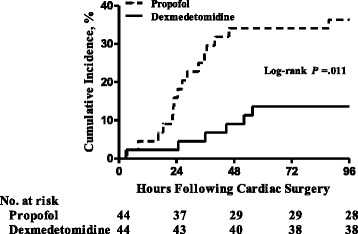
Kaplan-Meier incidence of postoperative atrial fibrillation according to treatment group

There were 22 patients who had AF during the first 96 h after cardiac surgery with CPB. Patients assigned to receive dexmedetomidine were significantly less likely to have AF than patients assigned to receive propofol (6 (13.6 %) vs 16 (36.4 %); odds ratio, 0.28; 95 % confidence interval, 0.10, 0.80; *P* = 0.025; number needed to treat, 4.4) (Table 2). The relative risk reduction was 62.6 %. The Kaplan-Meier incidence of postoperative AF in the dexmedetomidine group and propofol group is shown in Fig. 2. In patients who had received dexmedetomidine, the median onset of AF tended to be delayed compared with control patients (Table 2).

**Table 2:** Postoperative characteristics and resource utilization of the patient groups

Characteristics	Propofol (*n* = 44)	Dexmedetomidine (*n* = 44)	*P* value
AF during 96 h after surgery	16 (36.4)	6 (13.6)	0.025
Onset of AF (h after operation)	25.5 (19.5–36.4)	40.4 (19.3–53.1)	0.261
In-hospital mortality	1 (2.3)	0 (0)	>0.999
Mortality during study period	1 (2.3)	0 (0)	>0.999
Serum potassium before AF, mmol/L	4.2 (3.9–4.3)	4.0 (3.–4.1)	0.083
Peak postoperative cTnI, ng/mL	5.5 (2.9–9.5)	6.9 (2.6–11.8)	0.611
Hypotension	13 (29.5)	25 (56.8)	0.017
Bradycardia	1 (2.3)	4 (9.1)	0.360
Nausea/vomiting	10 (22.7)	9 (20.5)	>0.999
Delirium	5 (11.4)	0 (0)	0.055
Acute kidney injury	3 (6.8)	5 (11.4)	0.713
Mechanical ventilation ≥24 h	6 (13.6)	4 (9.1)	0.739
Intubation time, h	21.2 (17.0–22.8)	21.0 (16.9–22.5)	0.757
Length of ICU stay, days	3.5 (2.7–4.5)	2.9 (2.4–3.5)	0.008
Length of hospital stay, days	14.0 (11.0–17.0)	13.5 (11.3–17.0)	0.983
Postoperative hospital days	8.0 (7.0–10.0)	8.0 (7.0–9.8)	0.505
Hospital fees, Chinese yuan, ×10^4^	8.64 (7.32–9.63)	7.79 (6.72–9.05)	0.068

Data are presented as the number (%) or median (interquartile range). *AF* atrial fibrillation, *cTnI* cardiac troponin I, *ICU* intensive care unit

One patient in the propofol group died on the second postoperative day due to cardiac failure. There were no statistically significant differences between the groups in peak postoperative cardiac troponin I (cTnI), bradycardia, nausea and vomiting, mechanical ventilation ≥24 h, acute kidney injury, or delirium (Table 2). However, the incidence of hypotension was significantly higher in the dexmedetomidine group than in the propofol group (Table 2). Patients with hypotension recovered quickly after intravenous fluid replacement and/or adjusting the infusion speed of vasoactive drugs. The details of sedation and analgesia are reflected in Table 3.

**Table 3:** Details of sedation and analgesia in propofol and dexmedetomidine groups

	Propofol (*n* = 44)	Dexmedetomidine (*n* = 44)	*P* value
RASS at 4 h	-2 (-3 to -1)	-2 (-2 to -1)	0.488
VAS at 24 h	1.0 (1-2)	1.5 (1-2)	0.935
Extra analgesic requirements^a^	4 (9.1)	5 (11.4)	>0.999
Infusion speed at 4 h, mg/kg/h or μg/kg/h	0.98 (0.77 to 1.23)	0.68 (0.48 to 0.82)	
Total dosage, mg or μg	900 (625 to 1100)	572 (448 to 720)	
Infusion time, minutes	889 (645 to 1000)	870 (725 to 1019)	0.537

Data are presented as the median (interquartile range) or n (%). ^a^Number of patients who received extra analgesic treatment within 24 h after surgery. *RASS* Richmond agitation-sedation scale, *VAS* visual analog scale

Medical resource utilization is also reported in Table 2. Although the intubation time, length of hospital stay and postoperative hospital days were similar in the two groups, the length of ICU stay was significantly shorter in the dexmedetomidine group than in the propofol group (2.9 (2.4–3.5) vs 3.5 (2.7–4.5 days, *P* = 0.008), with a trend toward a decrease in median hospital fees (86,367 vs 77,874 Chinese yuan, *P* = 0.068). Patients who experienced postoperative AF were significantly older and had a longer ICU stay, more postoperative hospital days and higher hospital fees compared to patients without postoperative AF (Table 4).

**Table 4:** Comparison of patients with and without atrial fibrillation (AF) or flutter

Characteristics	Patients with AF (*n* = 22)	Patients without AF (*n* = 66)	*P* value
Age, years	62.5 (57.8–68.3)	52.0 (45.5–61.0)	<0.001
Female gender, *n* (%)	16 (72.7)	37 (56.1)	0.212
Body mass index, kg/m^2^	23.4 (20.9–27.1)	22.2 (19.8–24.0)	0.124
Cardiopulmonary bypass time, minutes	74.7 (61.0–96.0)	68.8 (55.2–85.1)	0.527
Cross-clamp time, minutes	48.5 (40.6–64.1)	48.2 (33.8–64.6)	0.643
Intubation time, h	21.2 (16.6–22.8)	21.0 (17.3–22.5)	0.528
Length of ICU stay, days	3.8 (2.9–4.8)	2.9 (2.4–3.7)	0.002
Length of hospital stay, days	15.0 (13.0–17.5)	13.0 (11.0–16.0)	0.117
Postoperative hospital days	9.5 (8.0–11.0)	8.0 (7.0–9.0)	0.021
Hospital fees, Chinese yuan, ×10^4^	8.91 (8.10–10.22)	7.80 (6.69–9.13)	0.006

Data are presented as the median (interquartile range) unless otherwise stated. *ICU* intensive care unit

## Discussion

To our knowledge, the current study is the first prospective randomized trial confirming that dexmedetomidine sedation reduces new-onset postoperative AF and shortens the length of ICU stay with a trend toward a decrease in hospital costs, compared to propofol sedation. The absolute risk reduction for AF was 22.8 % in patients following cardiac surgery, with a number needed to treat of 4.4, suggesting that dexmedetomidine administration during the early postoperative period could prevent one case of AF for every five patients. In addition, our results also demonstrated that postoperative AF was associated with a longer stay in the ICU, more postoperative hospital days and increased hospital fees.

Dexmedetomidine is an α_2_ adrenergic receptor agonist that induces sedative, anxiolytic and analgesic effects without causing respiratory depression [18, 29] and has been increasingly used for sedation in cardiac surgery patients [16, 29]. A small retrospective study (*n* = 45) reported that patients undergoing cardiovascular surgery who received dexmedetomidine were less likely to have new-onset AF than patients who did not receive any sedative drugs [22], which supports the results of the current study. However, a recent meta-analysis showed that dexmedetomidine was not associated with a decrease in the incidence of postoperative AF in patients undergoing cardiac surgery [30]. The potential reasons for this inconsistency may be the fact that postoperative AF was not the primary outcome, an explicit and consistent definition of AF was lacking, different observation periods were used for detecting AF, a large variety of types of surgery and drug regimens were included in those studies [31–36], and patients with prior AF were not excluded in most studies [31–35]. Some patients had a history of chronic, recurrent AF that was resistant to chemical cardioversion [18]. Consequently, the inclusion of patients with prior AF in the study may have concealed the efficacy of dexmedetomidine to reduce postoperative AF.

Several potential factors could contribute to the effect of dexmedetomidine on preventing new-onset postoperative AF. First, dexmedetomidine could reduce myocardial ischemia-reperfusion injury and improve the perfusion of the myocardium in patients undergoing cardiac surgery [16, 36]. Second, the inflammatory response induced by cardiac surgery and bypass can alter atrial electrophysiology and structural substrates, leading to increased vulnerability to AF [14, 37]. Dexmedetomidine has been shown to inhibit the inflammatory response in animal models [38, 39] and in clinical trials [40, 41]. Tasdogan et al. [41] found that postoperative patients who received dexmedetomidine had significantly lower levels of tumor necrosis factor-alpha, interleukin-1 and interleukin-6 than those who received propofol. Third, increased adrenergic tone plays a role in the development of postoperative AF [42]. Dexmedetomidine can decrease catecholamines [43] and inhibit the arrhythmogenic effect of epinephrine [44]. Fourth, dexmedetomidine can enhance vagal activity, leading to alterations in Ca^2+^ currents across the myocyte cell membrane, which in turn could result in prolonged repolarization and an increased effective refractory period [17]. Based on the combination of organ-protective, anti-inflammatory, sympatholytic and parasympathomimetic effects, it is not surprising that the use of dexmedetomidine during the early postoperative period could be beneficial in preventing AF after cardiac surgery.

An increased risk of bradycardia and hypotension can be a concern of dexmedetomidine [23, 24]. In this study, hypotension was more common among dexmedetomidine-treated patients despite the similar levels of sedation attained by patients treated with dexmedetomidine and propofol. However, hypotension was quickly rectified after intravenous fluid replacement and/or adjusting the infusion speed of the vasoactive or sedative drug, which rarely necessitated stopping the study in either group.

This study has several limitations. Fist, there was no blinding of the propofol and dexmedetomidine infusions in our study. However, the diagnosis of AF was made by continuous electrocardiogram (ECG) monitoring and/or 12-lead ECG, and the practitioners who identified AF were not aware of the study objectives.

Second, routine prophylactic strategies for postoperative AF, such as the perioperative use of β-blockers, were not implemented in our study. The choice of antiarrhythmic medications was left to the discretion of the attending physicians, but physicians chose drugs according to local guidelines. For example, digitalis was usually used to slow the ventricular rate during AF in our ICU. Furthermore, the attending physicians were also unaware of the study objectives. More patients received digitalis in the propofol group than in the dexmedetomidine group (see Additional file 1), which is mostly due to a greater number of postoperative AF incidents among patients receiving propofol.

Third, although the elderly patients were more likely to have postoperative AF, the patients in this study were younger than those in most other studies (mean age, 54 vs approximately 65 years) [1, 3, 31]. Moreover, only 16 patients in our study (18.2 %) were ≥65 years old. Therefore, additional studies are needed to investigate the effects of dexmedetomidine on the prevention of postoperative AF in elderly patients after cardiac surgery.

Fourth, tests about the secondary end points (e.g., the length of ICU stay) and the incidence of adverse events (e.g., hypotension) were performed without adjusting for multiple testing in this study. Therefore, these results are explorative and confirmatory studies are needed. However, our primary endpoint, the incidence of postoperative AF, was a single primary endpoint and therefore no adjustments are needed [45].

## Conclusions

Our results demonstrated that the use of dexmedetomidine might be efficacious in the prevention of postoperative AF and could be beneficial in reducing medical resource utilization in patients after cardiac surgery. Larger RCTs are needed to confirm our findings.

## Key messages

- Patients with new onset postoperative AF were significantly older and had a longer ICU stay, more postoperative hospital days and increased hospital fees
- Dexmedetomidine sedation reduced new-onset postoperative AF and shortened the ICU stay in patients after cardiac surgery compared to propofol sedation
- The administration of dexmedetomidine during the early postoperative period could prevent one case of AF for every five patients undergoing cardiac surgery

## Additional file

Additional file 1: Table S1. Postoperative medication use of the patients in propofol and dexmedetomidine groups. Description of data: medications were used within 96 h after cardiac surgery (DOCX 13 kb)

## Abbreviations

AF: Atrial fibrillation
AKIN: Acute Kidney Injury Network
BMI: Body mass index
CK-MB: Creatine kinase-MB
COPD: Chronic obstructive pulmonary disease
CPB: Cardiopulmonary bypass
cTnI: Cardiac troponin I
ECG: Electrocardiogram
ICU: Intensive care unit
LVEF: Left ventricular ejection fraction
MAP: Mean arterial pressure
NYHA: New York Heart Association
RASS: Richmond agitation-sedation scale
RCT: Randomized controlled trial
VAS: visual analog scale
