# Supplementary material for: Dexmedetomidine sedation reduces atrial fibrillation after cardiac surgery compared to propofol: a randomized controlled trial
Source: Crit Care. 2016 Sep 21;20:298. doi: 10.1186/s13054-016-1480-5 (PMC5031329; doi:10.1186/s13054-016-1480-5)
Supplement: Additional file 1: Table S1. — Postoperative medication use of the patients in propofol and dexmedetomidine groups. Description of data: medications were used within 96 h after cardiac surgery (DOCX 13 kb) [file 13054_2016_1480_MOESM1_ESM.docx]

**Additional file 1: Table S1** Postoperative medication use of the patients in propofol and dexmedetomidine groups

| Medications^*^ | Propofol (n = 44) | Dexmedetomidine (n = 44) | *P* value |
| --- | --- | --- | --- |
| Beta blocker, n (%) | 10 (22.7) | 14 (31.8) | 0.473 |
| Calcium channel blocker, n (%) | 15 (34.1) | 11 (25.0) | 0.484 |
| Amiodarone, n (%) | 7 (15.9) | 1 (2.3) | 0.058 |
| Digitalis, n (%) | 26 (59.1) | 15 (34.1) | 0.032 |
| Milrinone, n (%) | 24 (54.5) | 31 (70.5) | 0.186 |

^*^Used within 96 hours after cardiac surgery. Data are presented as a number (%)
